# Stabilizing the integrity of intestinal barrier to control arthritis

**DOI:** 10.1186/s13075-024-03378-7

**Published:** 2024-07-18

**Authors:** George D. Kalliolias, Athanasios G. Papavassiliou

**Affiliations:** 1 Hospital for Special Surgery, Arthritis & Tissue Degeneration, New York, NY 10021 USA; 2 Department of Medicine, Weill Cornell Medical College, New York, NY 10065 USA; 3 Regeneron Pharmaceuticals, Inc., Tarrytown, NY 10591 USA; 4 Department of Biological Chemistry, Medical School, National and Kapodistrian University of Athens, Athens, 11527 Greece

**Keywords:** HIF1a, RIPK3, Intestinal barrier, Necroptosis, Rheumatoid arthritis

## Abstract

With great interest, we have read the recent article “Expression of HIF1α in intestinal epithelium restricts arthritis inflammation by inhibiting RIPK3-induced cell death machinery” published by Lyu et al. in *Annals of the Rheumatic Diseases*. The authors pose that the expression of hypoxia-inducible factor 1 alpha in intestinal epithelial cells represents a crucial check point for the development of arthritis by impeding necroptosis of intestinal epithelial cells and safeguarding the intestinal barrier integrity. Previous studies suggest a potential mechanistic link between faulty intestinal barrier function and potentiation of arthritogenic immune cells. From this perspective, bolstering the intestinal barrier integrity arose as an attractive therapeutic strategy for rheumatoid arthritis.

## Dear editor

A recent study published in *Annals of the Rheumatic Diseases* suggests that the expression of hypoxia-inducible factor 1 alpha (HIF1a) in intestinal epithelial cells (IECs) represents a critical check point for the development of arthritis by inhibiting necroptosis of IECs and preserving the intestinal barrier integrity [1]. Prior studies indicate a potential mechanistic association between impaired intestinal barrier function and activation of arthritogenic immune cells [2–4]. In this context, strengthening the intestinal barrier integrity has emerged as an attractive therapeutic approach for rheumatoid arthritis (RA) [5].

**Fig. 1:**
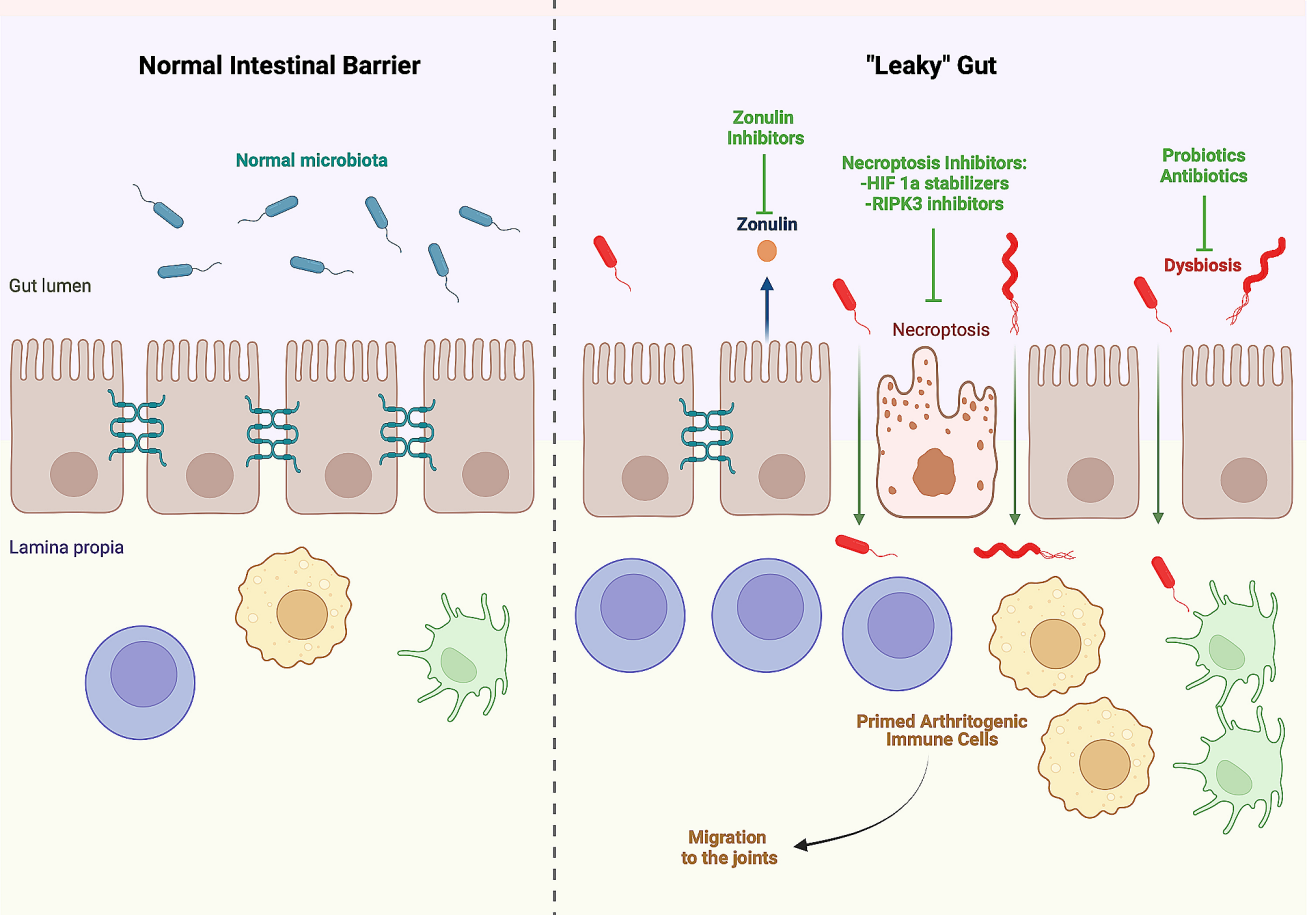
Normal intestinal barrier and the “leaky gut” of arthritis. The normal intestinal barrier is a physical barrier comprised of epithelial cells sealed by interepithelial tight junctions. In animal models, the onset of arthritis is preceded by dysbiosis and disruption of intestinal barrier integrity (“leaky” gut) due to secretion of zonulin by intestinal epithelial cells, destabilization of tight junctions, and necroptosis of epithelial cells. Inhibitors of necroptosis and antagonists of zonulin restore the integrity of intestinal barrier and ameliorate arthritis. HIF 1a, hypoxia-inducible factor 1 alpha; RIPK3, receptor-interacting protein kinase 3. This figure was created with BioRender.com

The intestinal barrier is a physical barrier comprised of continuously renewed specialized epithelial cells, sealed by interepithelial tight junctions (TJs) and a mucus layer covering the epithelium [5] (Fig. 1). TJs control the intercellular trafficking of toxins, antigens, and other microbial products from the gut lumen to the subepithelial mucosal layers and the bloodstream. TJs are assembled by a group of interacting proteins including zonula occludens (ZO)-1, claudins, and occludins [4]. In a series of animal models of arthritis (spontaneous arthritis in K/BxN mice, antigen-induced arthritis (AIA), and collagen-induced arthritis (CIA)) breach in intestinal permeability (“leaky gut”) has been identified using various functional assays [1–3]. Two independent animal studies in CIA have shown early evidence of “leaky gut” and bacteria translocation after mice immunization and even before the onset of arthritis [1, 2]. Three independent studies in humans have confirmed increased intestinal permeability in different stages of RA (i.e., pre-RA, early RA, and established RA) manifested by elevated serum levels of lipopolysaccharide (LPS), LPS-binding protein (LBP), intestinal fatty acid binding protein (I-FABP), and soluble CD14 (sCD14) [2, 3, 6].

Several distinct and potentially synergizing molecular mechanisms (summarized in Fig. 1) have been implicated in the breach of intestinal barrier integrity during the course of inflammatory arthritis. The recent study by Lyu et al. has identified death of intestinal epithelial cells via necroptosis as a critical mechanism of intestinal barrier disruption during the onset of CIA [1]. Transcriptomic analysis revealed upregulated expression of the necroptotic (receptor-interacting protein kinase 3 (Ripk3), Ripk1, and mixed lineage kinase domain like pseudokinase (Mlkl)) and apoptotic (caspase-3 and caspase-8) machinery in epithelial cells from the ileum of mice at the onset of CIA [1]. Other studies have revealed compositional and functional changes in gut microbiota (dysbiosis) and subclinical intestinal inflammation as possible drivers of TJ destabilization and “leaky gut” in animal models of arthritis and humans with RA [2, 3]. According to Tajik et al., gut dysbiosis directly triggers zonulin secretion by intestinal epithelial cells and induces zonulin-mediated downregulation of TJ proteins (ZO-1, occluding, and claudin-1) [2]. In addition, subclinical intestinal inflammation has been observed in treatment naïve patients with early RA as well as before the onset and during the course of arthritis in animal models, and there is evidence about an impact of inflammatory cytokines (such as tumor necrosis factor (TNF) and interferon gamma (IFNγ)) on the integrity of TJs by reducing the levels of occludins and ZO-1 [2, 5]. Finally, non-steroid anti-inflammatory drugs (NSAIDs), frequently used for pain control, have been suspected to disrupt the integrity of intestinal barrier and worsen gut leakiness in patients with arthritis [7].

One hypothesis for RA pathogenesis is that the disease begins in genetically predisposed individuals at mucosal sites (i.e., oral cavity, lung, gut) as a consequence of the interaction between a dysbiotic microbiota and the mucosal immune system, and then transitions to synovitis [8]. The temporal association of intestinal dysbiosis, subclinical gut inflammation, and “leaky gut” with the onset of arthritis in animal models may suggest a mechanistic link between increased intestinal permeability and RA pathogenesis in accordance with the “mucosal hypothesis”. In CIA, disruption of the integrity of intestinal barrier is associated with increased Th17 and Th1 infiltration of intestinal lymphoid organs and elevated serum levels of interleukin (IL)-17 and IFNγ [1]. Notably, pharmacologic restoration of the integrity of intestinal barrier resulted in reduced Th17 and Th1 infiltration and decreased serum levels of IL-17 and IFNγ, accompanied with amelioration of arthritis. In addition, a recently published study by Matei et al. has shown in patients with RA enrichment of peripheral blood mononuclear cells (PBMCs) in cells expressing the gut-homing markers LPAM-1 and CCR9 and the percentage of LPAM-1^+^CCR9^+^PBMCs was positively correlated with disease activity [3]. Pharmacologic inhibition of CCR9-mediated trafficking of these cells reduced the severity of arthritis in animal models, suggesting that recirculation of arthritogenic immune cells between the gut and the joints may contribute to the pathogenesis of RA [3]. Finally, another group has used a photoconvertible protein to track the trafficking of immune cells during the development of CIA and found primarily CD4 + T-cells migrating from the small intestine to the synovium, further supporting the concept of intestinal priming and recirculation of arthritogenic immune cells [2].

Taken together, these observations indicate a pathogenetic model where intestinal dysbiosis drives subclinical intestinal inflammation followed by disruption of intestinal barrier integrity due to epithelial cell necroptosis and destabilization of TJs (Fig. 1). Then, “leaky gut” allows unopposed entrance of microbial products from the gut lumen to the subepithelial mucosal layers and the bloodstream. These microbial products may work as antigens or molecular patterns that prime or stimulate arthritogenic immune cells which recirculate from the gut to the synovial joints triggering the onset of arthritis or fueling an already established synovial inflammation [4]. In this context, the anatomic and functional restoration of the intestinal barrier integrity may represent a promising therapeutic strategy for RA, especially for subjects that display inadequate response or toxicities to the standard of care immunosuppression.

Inhibition of intestinal epithelial cell necroptosis with HIF1a stabilizers or RIPK3 inhibitors [1] and strengthening the integrity of TJs with zonulin inhibitors or butyrate [2] have recently emerged as attractive therapeutic approaches to control arthritis (Fig. 1). The study by Lyu et al. identifies HIF1a and RIPK3 as critical players for the control of intestinal epithelial cell survival during the course of CIA [1]. HIF1a operates as a transcriptional repressor for the necroptosis-inducing RIPK3 in intestinal epithelial cells. Regulated proteasome degradation finetunes the protein levels of HIF1a depending on the tissue oxygen availability [9]. Under normoxic conditions, HIF1a is targeted for proteasomal degradation via hydroxylation of specific prolyl residues catalyzed by enzymes called prolyl hydroxylase domain proteins (PHD). Stabilization of HIF1a protein levels in intestinal epithelial cells with the PHD-inhibitor roxadustat or pharmacologic inhibition of RIPK3 with GSK-872 prevented necroptosis of intestinal epithelial cells and decreased the incidence and severity of CIA [1].

Larazotide acetate (AT-1001) is a synthetic octapeptide that acts as a zonulin antagonist that binds zonulin receptors. Treatment of mice with AT-1001 was sufficient to increase mRNA expression of TJ proteins, preserve the intestinal epithelial barrier integrity, block the migration of immune cells from the intestine to the joints, and reduce the severity of CIA [2]. Butyrate is a short-chain fatty acid (SCFA) produced by intestinal microbiota known for stabilizing intestinal TJs. Addition of butyrate in the drinking water of mice upregulated the expression of TJ proteins in intestinal epithelial cells, improved intestinal barrier function, and attenuated CIA [2].

Despite the promising evidence from preclinical models, the effectiveness of HIF1a stabilizers as a future treatment for RA requires careful evaluation due to the pleiotropic and cell-type specific effects of HIF1a in RA pathogenesis. Although HIF1a in intestinal epithelial cells is potentially therapeutic for arthritis due to its protective effect in intestinal barrier, in immune cells and synovial fibroblasts is arthritogenic [9]. Therefore, roxadustat that shows preferential distribution in the gastrointestinal tract subsequent to oral administration has promising tissue-targeting characteristics. Another issue requiring further investigation is whether stabilization of intestinal barrier represents suitable approach to prevent the development of full-blown RA in subjects with evidence of preclinical disease or to treat patients with established synovitis. Finally, given the role of intestinal dysbiosis as a driver for “leaky gut” and RA pathogenesis, a more detailed characterization of the RA-specific compositional and functional changes in gut microbiota is deemed necessary [10].

## Data Availability

No datasets were generated or analysed during the current study.

## Abbreviations

AIA: Antigen-Induced Arthritis
CIA: Collagen-Induced Arthritis
HIF1a: Hypoxia-Inducible Factor 1 alpha
IECs: Intestinal Epithelial Cells
I-FABP: Intestinal Fatty Acid Binding Protein
IFNγ: Interferon gamma
IL: Interleukin
LBP: LPS-Binding Protein
LPS: Lipopolysaccharide
Mlkl: Mixed lineage kinase domain like pseudokinase
NSAIDs: Non-Steroid Anti-Inflammatory Drugs
PHD: Prolyl Hydroxylase Domain proteins
RA: Rheumatoid Arthritis
Ripk3: Receptor-interacting protein kinase 3
sCD14: Soluble CD14
SCFA: Short-Chain Fatty Acid
TJs: Tight Junctions
TNF: Tumor Necrosis Factor
ZO-1: Zonula Occludens-1
